# Transcriptome analysis implicates secondary metabolite production, redox reactions, and programmed cell death during allorecognition in *Cryphonectria parasitica*

**DOI:** 10.1093/g3journal/jkaa021

**Published:** 2020-12-07

**Authors:** Anatoly A Belov, Thomas E Witte, David P Overy, Myron L Smith

**Affiliations:** 1 Department of Biology, Carleton University, Ottawa, ON K1S 5B6, Canada; 2 Agriculture and Agri-Food Canada, Ottawa, ON, K1Y 4X2, Canada

**Keywords:** allorecognition, transcriptomics, programmed cell death, heterokaryon incompatibility, secondary metabolites, RNA-seq

## Abstract

The underlying molecular mechanisms of programmed cell death associated with fungal allorecognition, a form of innate immunity, remain largely unknown. In this study, transcriptome analysis was used to infer mechanisms activated during barrage formation in *vic3*-incompatible strains of *Cryphonectria parasitica*, the chestnut blight fungus. Pronounced differential expression occurred in barraging strains of genes involved in mating pheromone (*mf2-1*, *mf2-2*), secondary metabolite production, detoxification (including oxidative stress), apoptosis-related, RNA interference, and HET-domain genes. Evidence for secondary metabolite production and reactive oxygen species (ROS) accumulation is supported through UPLC-HRMS analysis and cytological staining, respectively. Differential expression of mating-related genes and HET-domain genes was further examined by RT-qPCR of incompatible interactions involving each of the six vegetative incompatibility (*vic*) loci in *C. parasitica* and revealed distinct recognition process networks. We infer that vegetative incompatibility in *C. parasitica* activates defence reactions that involve secondary metabolism, resulting in increased toxicity of the extra- and intracellular environment. Accumulation of ROS (and other potential toxins) may result in detoxification failure and activation of apoptosis, sporulation, and the expression of associated pheromone genes. The incompatible reaction leaves abundant traces of a process-specific metabolome as conidiation is initiated.

## Introduction

Programmed Cell Death (PCD) in filamentous fungi is involved in a variety of processes including intraspecific mycelial incompatibility, aging, and spore formation (Raju and Perkins 2000; Barhoom and Sharon 2007) and demonstrates morphological features of apoptotic death (Glass and Kaneko 2003; Dementhon *et al.* 2006). The pathways that lead to PCD in fungi are not clearly identified (Shlezinger *et al.* 2012) but may be unraveled through studies on mycelial incompatibility systems that are referred to as heterokaryon incompatibility (HI) in *Podospora anserina* and *Neurospora crassa* and vegetative incompatibility (VI) in *Cryphonectria parasitica*. In these ascomycete species, HI and VI are genetically determined by 6–12 incompatibility loci and polymorphisms at these loci create the basis for conspecific nonself recognition (Glass and Dementhon 2006; Paoletti and Saupe 2009; Smith and Lafontaine 2013). Hyphal fusion of two members of the same species that possess different alleles at one or more vic or het loci results in an incompatibility reaction in the fusion cells and subtending cells that progresses from cytoplasmic granulation and vacuolization to plasmolysis and cell death. In most cases, known *vic* (and *het*) loci contain two or more tightly linked genes, at least one of which encodes an HET domain (IPR010730) (Glass *et al.* 2000; Smith *et al.* 2000; Paoletti and Clavé 2007; Smith and Lafontaine 2013). Although the molecular function of the HET domain remains unclear, an association exists between the HET domain and fungal nonself recognition, where transcriptional activation of HET-domain genes correlates with activation of PCD (Paoletti *et al.* 2007). Fungal genomes can carry over 50 HET-domain genes. However, in any given genome, only a small subset of the genes containing HET domains have been associated with conspecific incompatibility and general allorecognition (Paoletti and Clavé 2007).

There are six characterized *vic* loci associated with VI in *C. parasitica* and each locus comprises at least two linked genes that interact to modulate nonself recognition (Choi *et al.* 2012; Zhang *et al.* 2014). Molecular genetic analyses indicate that the *vic1*, *vic6*, and *vic7* loci all possess genes with a HET domain whereas *vic2*, *vic3*, and *vic4* do not (Zhang *et al.* 2014). Therefore, it may appear that an HET domain is not a necessary component for VI reaction, at least in *C. parasitica*. However, in some systems, incompatibility loci are known to interact with unlinked HET-domain genes. For example, in *N. crassa*, HI is triggered by differences at the *mat* locus only when the unlinked *tol* gene (contains HET and leucine-rich repeat domains) is expressed (Shiu and Glass 1999). When *tol* is deleted, strains of opposite mating types are able to form viable heterokaryons during the vegetative phase of the life cycle. When *tol* is present, cells containing *mat-A* and *mat-a* exhibit HI during vegetative growth. Thus, incompatibility loci that do not contain genes with an HET domain may be functionally coupled with unlinked genes that do have the HET domain that do not necessarily show genetic polymorphism in incompatible strains.

In this study, we analyze transcription profiles to identify genes that are differentially expressed (DE) during *vic3*-associated nonself recognition in *C. parasitica*. We provide general observations on overall expression patterns, perform functional annotations on DE genes using gene databases, and enrichment analyses in order to understand the underlying mechanisms involved in allorecognition in this fungus. The analysis of DE genes showed activation of mating pheromone genes, apoptotic-like factors, and indicate the involvement of reactive oxygen species (ROS) and secondary metabolite biosynthesis pathways in allorecognition processes. These patterns were confirmed with secondary assays based on quantitative RT-PCR, microscopy using redox-sensitive stains and metabolomic profiling by mass spectrometry.

## Materials and methods

### Strains and growth conditions

*Cryphonectria parasitica* strains P74-3, EP155, and DZ-66 were used for transcriptome analysis in this study. Strains P74-3 and EP155 are of a distinct genetic background (Biella *et al.* 2002), but carry identical alleles at all *vic* loci except for *vic3*. The EP155 strain carries the *vic3-1* haplotype and P74-3 carries the *vic3-2* haplotype. Strain DZ-66 is derived from EP155 where genes *vic3a-1* and *vic3b-1* were knocked out (Zhang *et al.* 2014). Additional *C. parasitica* strains used in this study are listed in Supplementary Table S1.

Strains were inoculated into 12-ml liquid of 2% potato dextrose broth (PDB, BD Difco Brand, NJ, USA) and incubated static for 7 days at 30°C in the dark. They were then diluted 10 times with fresh 2% PDB and blended at high speed. We reasoned the maceration of mycelia into small fragments maximizes the number of contacts formed when strains are co-inoculated together, thereby amplifying transcriptomic and metabolomic signals associated with incompatibility. The resulting cell suspensions had approximately 2000 Colony Forming Units (CFUs) per ml. Three strains (EP155, P74-3, DZ-66) were used as “monoculture” controls. The mixed culture EP155 + P74-3 represented the *vic3*-incompatible (barraging) interaction while DZ-66 + P74-3 was used as a *vic*-compatible control.

Cultures for RNA extraction were grown in 8.5-cm Petri plates on potato dextrose agar (PDA, BD Difco Brand, NJ, USA) overlaid with sterile semipermeable cellophane membranes. Approximately 200 CFUs (100 µl) of each hyphal suspension was evenly spread over the membrane. Cultures were incubated in the dark at 30°C for 3 days, at which point all mycelium was removed from the membrane (approximately 3 g per sample), frozen with liquid nitrogen, and stored at −80°C. Three biological replicates were plated for RNA extraction, and six biological replicates were plated for metabolite extraction.

### RNA preparation and analysis

Total RNA was extracted using the Qiagen RNeasy Plant RNA extraction kit following the manufacturer manual (Qiagen, Valencia, CA, USA) with a DNAse treatment step. RNA quality was assessed with Agilent Bioanalyser (Santa Clara, CA, USA). RNA sequencing was done on Illumina NovaSeq 6000 platform, with paired-read length of 150 bp and 70 M read depth (Genome Quebec, Montreal, Canada).

### RNA-seq data analysis

Short reads quality was assessed with FastQC and reference-based alignment was performed with STAR 2.7 (Dobin *et al.* 2013). Reference genome and functional annotation and gene mapping were acquired from the JGI *C. parasitica* genome portal (https://mycocosm.jgi.doe.gov/Crypa2/Crypa2.home.html), which contains information about 11,609 *C. parasitica* protein-coding areas (Crouch *et al.* 2020). Additionally, novel transcripts from regions of the genome that showed significant expression, but were not previously annotated as genes were identified using StringTie v.2.1.3 (Pertea *et al.* 2015). Transcripts were identified using alignment data obtained from STAR alignment. Identified transcripts showing counts less than 10 were discarded. As a result, 84 novel transcripts were identified, for a total of 11,693 genes. Differential expression analysis was done with the R package DESeq2 (Love *et al.* 2014) and further, data analysis and visualization were done in the R environment. Gene Enrichment analysis was done using DAVID v6.7 (Huang *et al.* 2009; Huang da *et al.* 2009). The significance threshold for gene differential expression was set to LFC (log2|fold change|) ≥|2| and FDR adjusted *P*-value <0.001.

### Analysis of intracellular ROS

ROS production during *vic3* barrage was assessed using the previously described microscopy protocol by Biella *et al.* (2002) coupled with DCFDA (2ʹ,7ʹ-dihydrodichlorofluorescein diacetate, Sigma-Aldrich, ON, Canada) staining. Inoculum blocks of 0.5 mm 3 were taken from the edges of cultures grown for 7–10 days on PDA. These agar cubes with mycelium were placed approximately 1 cm apart on microscope slides coated with PDA. Slides were incubated in a damp chamber for 2 days in the dark at room temperature, after which inoculum agar cubes were removed and a stained with DCFDA (10 µM) (LeBel *et al.* 1992; Hutchison *et al.* 2009). ROS accumulation oxidizes DCFDA to fluorescent DCF and barraging cells were observed with fluorescent microscopy (Carl Zeiss, AxioVision).

### RT-qPCR conditions

Reverse transcription from RNA template was performed using M-MuLV Reverse Transcriptase (New England Biolabs, Whitby, ON, Canada) according to manufacturer recommendations. Real-time quantitative PCR analysis was performed using a CFX Connect Real-Time PCR Detection System (BioRad, Mississauga, ON, Canada) with KAPA SYBR FAST Universal 2× master mix (KAPA, Wilmington, MA, USA). Gene expression values were normalized against 18S rRNA. Relative abundance of normalized transcripts was calculated using $2^{-\Delta \Delta Ct}$ method (Livak and Schmittgen 2001). Real-time PCR primers used for measuring transcript abundance of selected *C. parasitica* genes are given in Supplementary Table S2.

### Liquid chromatography-mass spectrometry analysis

Mycelium and the agar directly underneath the membrane were harvested into glass scintillation vials as separate samples from each plate after 5 days of incubation in the dark at 30°, in six replicates, along with media controls (PDA with sterile membrane overlaid) and immediately frozen at −20°. Thawed samples were extracted by immersion in ethyl acetate (gently shaking for 1.5 h), dried under vacuum, and then reconstituted in MeOH to a concentration of 500 µg/ml. The ultra-high performance liquid chromatography coupled high resolution mass spectrometry (UPLC-HRMS) analysis was carried out on a Thermo Ultimate 3000 UPLC coupled to a Thermo LTQ Orbitrap XL high-resolution mass spectrometer, using a reverse-phase Phenomenex C18 Kinetex column in ESI+ mode (with an *m/z* 100–2000 *m/z* range). Data preprocessing closely followed the methodology of Overy *et al.* (2017), using MZMine v2.29 [Cell Unit, Okinawa Institute of Science and Technology (OIST), Onna, Okinawa, Japan], with a mass detection noise cutoff level set to $5.0\times 10^{5}$. Mass features (each representing an associated retention time and mass/charge ratio) from agar and mycelium extracts were normalized to the total ion current detected from each sample and then summed, scaled, and centered.

### Data availability

Supplementary files are available at G3 FigShare. File “Belov_etal_TableS1_2.pdf” contains information about *C. parasitica* strains used and qPCR primer sequence information. Supplementary File “Belov_etal_TableS3.xlsx” contains data for Figure 3 (heatmap). Table 1 and Supplementary Table S3 contain data for gene differential expression with annotations. Supplementary “Belov_etal_TableS4.xlsx” contains a list of orthologs DE in *C. parasitica* during *vic3* associated barrage and *N. crassa* and *P. anserina* during HI. R scripts used in this work can be found on https://github.com/anabeloff/vic3PCD.

**Table 1:** Notable genes and their differential expression during *vic3*-associated barrage formation (*P*-values > 0.001 shown grey)

Groups	**Cp ID** ^a^	**LFC** ^b^	*P*-value	UniProt ID	Putative protein function
	66954	6.1	2.52e−16	P55211	Weak similarity to human Caspase-9
	75073	5.1	1.27e−69	Q9VFP2	Protein roadkill (*rdx*)
	333952	3.4	4.22e−12	Q53FA7	Quinone oxidoreductase (QO)
Apoptosis	259069	3.3	9.58e−206	Q15392	3-beta-hydroxysterol delta-24-reductase (DHCR24)
	258862	2.4	1.97e−20		Heterokaryon incompatibility (*vic1a*)
	262887	2.3	1e−102	Q9UV10	Heterokaryon incompatibility (*dev3-2*)
	261856	2	1.29e−14	Q9UV10	Heterokaryon incompatibility (*dev3-1*)
HET	67224	−1.1	3.08e−11	Q9C2N1	Transcription factor *vib-1*
	85578	12.9	2.63e−116	O14431	Mating pheromone precursor *mf2-1*
	CPNOV.6992^c^	10.4	8.88e−251	O14431	Mating pheromone precursor *mf2-2*
	44005	7.1	1.01e−51	P35693	MAT-2
	96416	5.6	0	P12866	Mating factor A secretion protein STE6
	262923	3.9	5.21e−12	A9CPT4	Tudor domain-containing protein 1
	351932	3.6	1.54e−272	P36631	Transcription factor *ste11*
	285012	2.8	2.21e−109	P08965	Meiosis protein, *mei2*
Mating	66950	0	0.767	P23561	NRC1 ortholog
	58765	6.9	0	Q9Y7Q2	Glutathione S-transferase
	274617	4.6	3.49e−66	Q64505	Cytochrome P450
	269746	4.6	4.14e−229	Q27712	Cytochrome P450
Oxidative stress	332509	−2.4	6.68e−06	Q4WY82	Psi-producing oxygenase A (*ppoA*)
Pigment	263100	8.9	2.67e−227	Q9F723	Hydroxyneurosporene dehydrogenase
RNAi	261854	2.8	9.98e−15	O74957	Argonaute-like protein 4 (*agl4*)
	345802	4.4	1.33e−104	Q9Y8G7	Cytochrome P450
	342071	4	1.75e−103	Q00714	P450 monooxygenase
	339678	2.2	8.73e−14	Q12609	P450 monooxygenase
	255335	−1.9	4.5e−09	Q4WAW6	O-methyltransferase
Sec. Metabolite Biosynthesis	18749	−2.9	5.85e−11	Q00668	Peroxidase

a Proteins IDs of *C. parasitica* genome v2 from JGI.

b LFC—Log2|fold change|.

c CPNOV—Novel transcripts identified in this study and not annotated in *C. parasitica* genome v2.

Supplementary material is available at figshare DOI: https://doi.org/10.25387/g3.8239778.

## Results and discussion

### Fungal anastomosis and cell death

**Figure 1:**
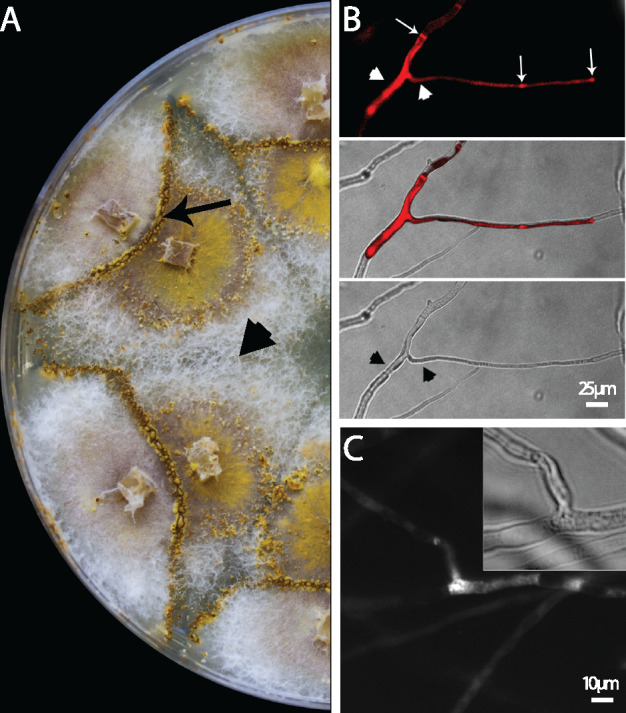
Cell death and ROS accumulation during *vic3*-associated barrage formation. (A) Barrage formed at the confluence of the *vic3*-incompatible *C. parasitica* strains EP155 (*vic3-2*) + P74-3 (*vic3-1*) is indicated by an arrow. No barrage is evident with self-pairings (e.g., P74-3 + P74-3, arrowhead). (B) Micrographs show that hyphae undergoing incompatible fusions fluoresce under UV light (arrowheads, top panel) due to accumulation of Evan’s Blue in dying/dead cells. Incompatible hyphae are compartmentalized by septa (narrow white arrows). Morphological changes in cell structure can also be seen under bright field illumination (bottom panel). The middle panel is an overlay of top (UV) and bottom (bright field) panels. (C) Brightfield (inset) and fluorescent micrographs show ROS production during *vic3*-incompatible hyphal fusions. Strains EP155 and P74-3 were grown for 2 days on microscope slide and stained with DCFDA. Fused cells from incompatible strains produce bright fluorescence as a result of ROS production. Self-pairings and P74-3 + DZ66 pairings do not show ROS signals (not shown).

Allorecognition following incompatible hyphal fusions is associated with rapid activation of PCD in *C. parasitica* and other filamentous ascomycetes (Jacobson *et al.* 1998; Biella *et al.* 2002). This process can be observed macroscopically when the *vic3*-incompatible strains (P74-3 *vic3a-2 vic3b-2 *+* *EP155 *vic3a-1 vic3b-1*) are co-inoculated on agar plates (Figure 1A). In these pairings, a barrage zone appears where the two mycelia meet, characterized macroscopically by a clear line that is flanked by a region with pigment production and active sporulation. We confirmed that barrage zones do form with pairings of P74-3 + EP155 but not in pairings of P74-3 + DZ66 (Δ*vic3a-1* Δ*vic3b-1*). Hyphal anastomosis of VI strains was observed microscopically with the Evans Blue assay, where hyphal fusion results in dead cells that are unable to actively transport dye out of the cell and thus become brightly fluorescent (Figure 1B). ROS accumulation was also observed at the site of hyphal fusion only in pairings of P74-3 and EP155 (Figure 1C).

### Hierarchical cluster and enrichment analyses

**Figure 2:**
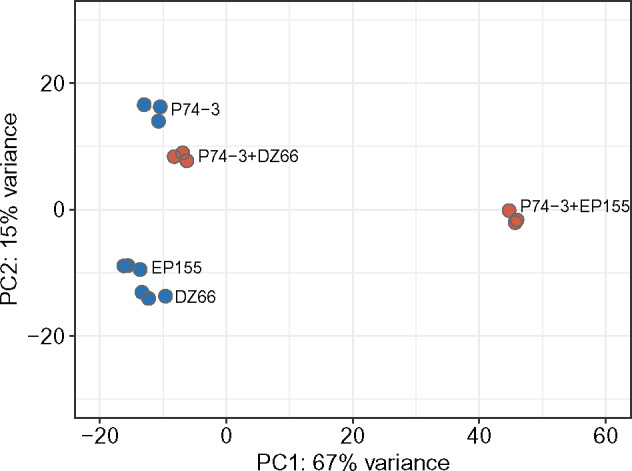
PCA of normalized expression counts of monoculture (blue) and mixed (red) samples. Mixed samples with *vic3* knockout strain (P74-3 *vic3a-2 vic3b-2 *+* *DZ66 Δ*vic3a-1* Δ*vic3b-1*) cluster together with monoculture samples along PC1. The plot demonstrates highest variance in gene expression between barrage sample P74-3 + EP155 *vic3a-1 vic3b-1* and control samples.

**Figure 3:**
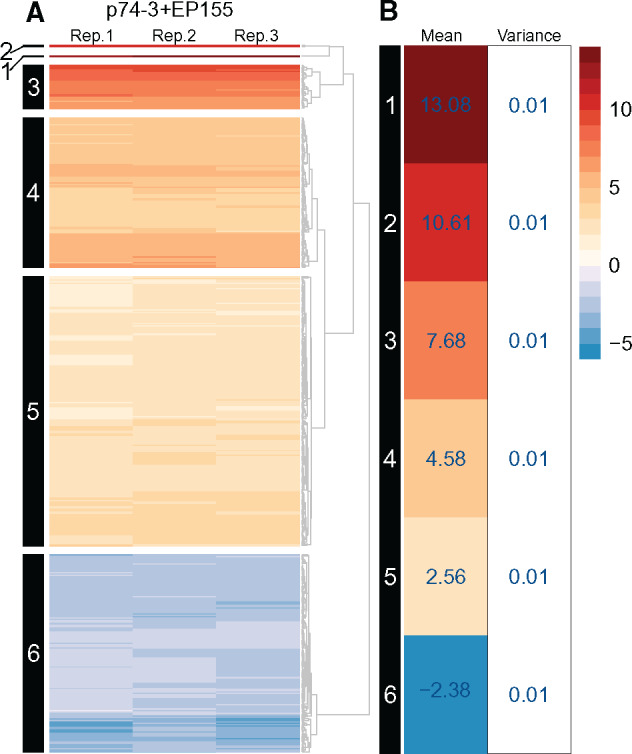
Hierarchical clustering of 531 DE genes (LFC≥|2|, P-value<0.001). (A) Estimated Euclidian distance between expression values was clustered with UPGMA (Unweighted Pair Group Method with Arithmetic Mean) for each of three replicate experiments. Cluster numbers are given at left. (B) Mean expression for each cluster (number at left within panel). Heat map was arranged according to mean expression in individual clusters. Notable genes in each cluster are presented in Table 1, complete cluster compositions are shown in Supplementary Table S3.

Principal component analysis (PCA) revealed differences in overall gene expression patterns between *vic3*-incompatible strain pairings (P74-3 + EP155), control pairing (P74-3 + DZ66), and monocultures (P74-3, EP155, or DZ66 alone) (Figure 2). Samples formed two distinct clusters, one of monocultures grouped close together with the control pairing (P74-3 + DZ66) and a second of *vic3*-incompatible strain pairings (P74-3 + EP155). Separation between barraging and control samples accounted for 70% of variance in the sample set. Significant (over 50%) variance provided evidence of differential gene expression between the two clusters. Differential expression analysis was performed using the DESeq2 package to obtain a list of 531 (501 genes + 30 novel transcripts) most DE genes based on the set threshold (LFC≥|2|, P−value<0.001). Using hierarchical clustering, we arranged these 531 DE genes in clusters based on individual LFC values (Figure 3, for detailed description of each cluster, see Supplementary Table S3). Six robust clusters were identified with a majority of DE genes (70%) upregulated during allorecognition. Downregulation was not a notable feature of barraging strains, evident only in cluster 6 (Figure 3). Clusters 5 and 6 comprise genes with mean LFC rates close to the threshold (LFC ≥|2|), making interpretation of activation or deactivation of these genes inconclusive. Thus, using clustering analysis, we narrowed down the list of possible candidate genes involved in barrage formation and associated PCD within clusters 1–4.

**Figure 4:**
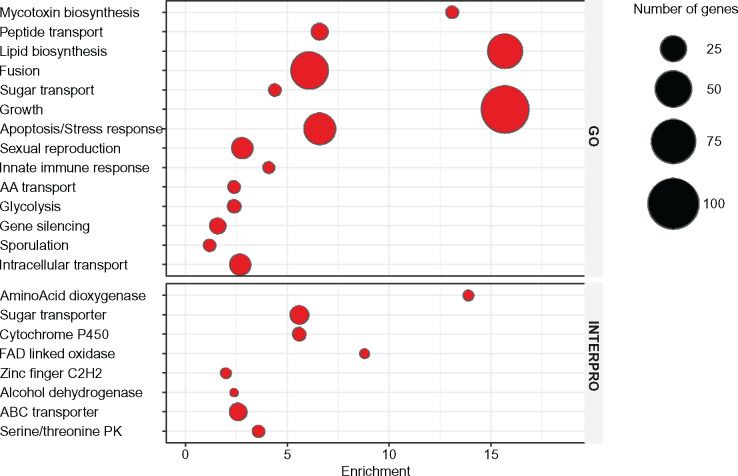
Gene enrichment analysis of GO terms for biological process and INTERPRO domains. Similar GO terms and INTERPRO domains were grouped together by DAVID using Kappa statistic. For GO term enrichment group, names were assigned based on types of GO terms in each group. Enrichment estimated as a ratio of genes in DE list associated with the term (GO or INTERPRO) to the total number of genes associated with the term in the background list.

Gene enrichment analysis included two main elements: a list of DE genes and a background list, against which enrichment of Gene Ontology (GO) annotation terms and protein domains were estimated (Figure 4). A total of 8031 *C. parasitica* gene IDs with UniProt annotations were used as a background list. For this analysis, we used annotation terms to form gene enrichment groups. In Figure 4, GO terms and INTERPRO domains were grouped together based on their functional similarity and then an enrichment score was calculated as an average ratio of the term among DE genes versus number of genes associated with the term in the background list. Using the above techniques, we distinguish five functional gene groups that represent processes playing major roles during barrage formation. The groups are designated as mating, apoptosis, HI, secondary metabolism, oxidative stress and detoxification, and RNA interference.

### Transcriptome analysis—mating

In *C. parasitica*, there are two mating types, MAT-1 and MAT-2. Each haploid strain carries a single mating-type transcription factor gene, *mat-1* or *mat-2*, but carries all three mating pheromone genes (*mf1-1*, *mf2-1*, *mf2-2*) (Kazmierczak *et al.* 1996; Zhang et al. 1998; Marra and Milgroom 2001). Expression of pheromones depends on what mating type is present in the haploid mycelium. Strains expressing MAT-2 activate *mf2-1* and *mf2-2* pheromone precursor genes, while ones with MAT-1 activate *mf1-1*. Strains used in our RNA-seq experiment, P74-3, EP155, and DZ66, are all MAT-2. In the RNA-seq analysis, the *mat-2* gene and associated pheromone genes *mf2-1* and *mf2-2* show the most dramatic change in expression of any genes during barrage (Figure 3, Cluster 1, Table 1).

Involvement of mating genes in barrage processes was unexpected and merited closer examination. We identified additional DE orthologs that were previously connected to mating in yeast. In cluster 4, we find genes *cpste6* and *cpste11* (orthologs of Ste11 associated with reproduction) that were overexpressed in *vic3*-incompatible barraging cultures. There are two yeast genes identified as *Ste11* in the literature. One encodes MAP kinase Ste11p, a homolog of *N. crassa* NRC1 (*cpnrc-1*, Cp ID: 66950), which is a component of the fusion oscillation complex (Pandey *et al.* 2004; Dettmann *et al.* 2014). The second gene identified as Ste11 (Cp ID: 351932, *cpste11*) is an ortholog of the *Schizosaccharomyces pombe* transcription factor Ste11p. *cpste11* gene orthologs belong to a family of high-mobility group transcription factors involved in conjugation and activation of the meiotic cycle (Qin *et al.* 2003). In *S. pombe*, Ste11p is activated in response to starvation or mating pheromones. As a transcription factor, Ste11p regulates the expression of several genes related to yeast mating, which include mating genes, pheromones, and meiosis regulatory factors (Kjaerulff *et al.* 1997; Kitamura *et al.* 2001; Mata and Bähler 2006). In our dataset, we identified two overexpressed orthologs of yeast genes that are known to be regulated by Ste11p. The first of these genes is a meiotic factor *mei2* ortholog (ID: 285012) that was upregulated during barrage. The second is *Ste6* (*cpste6*, Cp ID: 96416) that encodes an ABC-transporter responsible for secretion of a-factor (Sugimoto *et al.* 1991; Kolling and Hollenberg 1994). Overexpression of *cpst6* and MAT-2 pheromones could indicate that barraging cells increase pheromone production and pump them out with *cpst6*, while other cells react to extracellular pheromone signal and activate the Ste11 system.

### Transcriptome analysis—RNA interference

In analyzing enriched genes associated with sexual reproduction, we identified a homolog of the fruitfly tud gene. TUD carries a Tudor domain and works as an essential part of the nuage structure, activated during germline cell development. This structure employs PIWI, an RNAi complex of proteins that prevents activation of retrotransposons (Kibanov *et al.* 2011). That *cptud* (ID: 262923) is overexpressed may indicate a role for RNAi during barrage in *C. parasitica*.

According to our RNA-seq analysis, the Argonaute-4 ortholog (previously identified in *C. parasitica* as *agl4*) (Sun *et al.* 2009) was overexpressed during barrage (Cluster 5); the RNAi specialization of *agl4* has yet to be confirmed. Among four Argonautes (*agl1*, *agl2*, *agl3*, and *agl4*) and two Dicer genes (*dcl1* and *dcl2*) in *C. parasitica*, only the RISC complex formed by Dicer-2 and Argonaute-2 was found to function as an antiviral defence, where expression of *agl2* is important to activate expression of *dcl2* (Zhang and Nuss 2008; Sun *et al.* 2009). Others, Dicer-1 and Argonauts 1, 3, and 4, were not found to be associated with known RNAi processes. None of *agl1*, *agl2*, *agl3*, *dcl1*, nor *dcl2* were DE during barrage. An increase in Argonaute expression should be followed by an increase in Dicer expression (Sun *et al.* 2009); therefore, it is possible that the observed overexpression of *agl4* during the initial onset of barrage represents an early stage of RNAi activation and that activation of Dicer will follow.

### Transcriptome analysis—HI

There are 124 ORFs annotated with an HET domain in the *C. parasitica* genome v.2. Even though HET-domain genes are associated with barrage formation, only three are linked to *vic* loci in *C. parasitica*. While genetic polymorphism in HET-domain genes is associated with VI in fungi (Smith *et al.* 2000), the majority of examined HET-containing ORFs lack genetic polymorphism among *C. parasitica* strains (Choi *et al.* 2012; Zhang *et al.* 2014).

Our analysis demonstrates that *vic3* incompatibility triggers upregulation of three HET-domain genes (*P*-value < 0.001) that are unlinked to the *vic3* locus. Surprisingly, one of these HET-domain genes, *vic1a* (Cp ID: 258862), is a component of the *vic1* locus and is also associated with barrage formation when strains carry different alleles at *vic1* (Zhang *et al.* 2014). Since the *vic3*-incompatible strains used in this experiment are identical at the *vic1* locus, we infer that differences at *vic3* act in trans to activate *vic1a*. The other two upregulated HET-domain genes were not previously associated with incompatibility and have not been assigned genetic designations. We refer to these genes as *dev3-1* (Cp ID: 261856) and *dev3-2* (Cp ID: 262887).

### Transcriptome analysis—apoptosis

Overall, the observation of enrichment in the “Apoptosis/Stress response” GO group indicates that PCD processes involved in *vic3* incompatibility are most likely activated by external signaling. This group of transcripts comprises GO terms associated with positive and negative regulation of apoptosis. In P74-3 + EP155 mixed-culture plates, it is expected that both positive and negative regulation of apoptosis would be occurring simultaneously; positive regulation in barraging cells as evidenced by cell death, and negative regulation (likely related to defence mechanisms) occurring in nearby cells that are not directly involved in incompatible cell fusions.

An example of an upregulated positive regulator of cell death was gene Cp ID: 75073, an ortholog of Protein roadkill (*rdx*). Similar to AIF (Apoptosis-inducing factor), *rdx* orthologs are involved in the extrinsic cell death pathway, as they are known to activate the JNK-dependent apoptotic cycle (Liu *et al.* 2009). As demonstrated in animals, JNKs are part of an external stress-activated kinase cascade, which leads to apoptosis through the activation of PCD transcription factors like p53 (Davis 2000; Yin *et al.* 2000). To activate the downstream PCD pathway, JNK must be activated by other protein kinases (PKs). Studies showed that an ortholog of yeast Ste20 in animals is responsible for activation of JNK (Brown *et al.* 1996). Ste20 belongs to a group of PKs called PAK (p21-activated serine/threonine kinases) that are upstream activators of the MAPK cascade (Dan *et al.* 2001). Orthologs of Ste20 were not among the differentially regulated genes observed during *vic3*-associated incompatibility.

Of additional interest within the “Apoptosis/Stress response” group was the upregulated Caspase-9 gene (Cp ID: 66954). It has low identity to human Caspase-9 and prediction of gene function in this case is difficult to interpret due to low sequence homology. Furthermore, transcript upregulation of fungal metacaspases is not necessarily an indicator of increased protein activity and apoptosis (Tsiatsiani *et al.* 2011). As a hallmark of the classic apoptosis pathway, the caspase cascade is activated primarily by post-translational modifications and increased accumulation of transcripts is not an expected outcome (Riedl and Salvesen 2007). Accordingly, previous studies on *N. crassa* showed no differential caspase expression during HI (Hutchison *et al.* 2009). Similarly, the other three predicted *C. parasitica* metacaspases show no significant change in expression during *vic3*-associated incompatibility.

Lastly, the “Apoptosis/Stress response” group contains genes that function to delay apoptosis onset. Gene 333952, an ortholog of a negative regulator of apoptosis QO (Quinone Oxidoreductase) was found to be differentially upregulated in incompatible mixed cultures. The activation of QO may be a response by cells near barraging cells in the mixed cultures. In animals, QOs are part of the cell defence mechanism that is activated in response to abiotic stress factors to maintain the cell’s redox balance (Johnson *et al.* 2008). Similarly, gene 259069, an ortholog of human DHCR24 (3*β*-hydroxysterol Δ24-reductase), was also upregulated during incompatibility. In humans, this gene is part of the cholesterol biosynthesis pathway and determines resistance to apoptosis caused by abiotic stress, where increased expression of DHCR24 leads to more resistance to apoptosis caused by ROS and other abiotic factors (Di Stasi *et al.* 2005; Kuehnle *et al.* 2008).

### Transcriptome analysis—oxidative stress and detoxification

The transcription of multiple genes relating to oxidative stress and detoxification was found to be upregulated in barraging strains (Table 1 and Supplementary Table S3) and corresponds with ROS production and accumulation that is observed at the site of incompatible hyphal fusions (Figure 1C). Three Glutathione S-Transferase (GST) paralogs (where ID 58765 was highly overexpressed) and an ortholog of QOs were activated during barrage formation and relate to cellular detoxification; GST is a member of the Phase II detoxification program and QOs are part of the antioxidant response system in animal cells (Johnson *et al.* 2008). GSTs and QOs are transcriptionally regulated by the Nrf2 transcription factor that activates a battery of anti-stress enzymes (including increases in QO and GST expression) in response to ROS-induced redox imbalance and elevated toxicity due to toxin exposure (Jaiswal 2000; Johnson *et al.* 2008; Ray *et al.* 2012; Tew and Townsend 2012). GSTs bind glutathione to toxic molecules, making them more accessible for transport out of the cell and QOs deactivate toxic quinone derivatives (Jaiswal 2000). Glutathione balance within the cell is pertinent to survival and dramatic reductions of glutathione levels due to high toxic exposure may lead to apoptotic death (Circu and Aw 2012).

One of the highly enriched INTERPRO domain terms relates to cytochrome P450 proteins responsible for phase I detoxification (see Figure 4). Activation of these genes is strongly correlated with toxic environmental stress and often used as a diagnostic marker of oxidative stress (Uno *et al.* 2012). For example, we identified upregulation of the ortholog (Cp ID: 345802) of cytochrome p450foxy from *Fusarium oxysporum*, an example of a self-sufficient p450 monooxygenase that is capable of functioning without the aid of an external p450 reductase (Kitazume *et al.* 2000). Cytochrome p450foxy and its analogs are believed to be responsible for denitrification performed by various fungi (Shoun and Takaya 2002).

During allorecognition in *C. parasitica*, expression of a lipoxygenase ortholog ppoA (Cp ID: 332509) was significantly decreased in barraging strains. Downregulation of lipoxygenase orthologs (*P. anserina* ID: Pa_5_1240) during HI in *P. anserina* has been previously reported (Bidard *et al.* 2013). Tsitsigiannis *et al.* (2005) suggest that downregulation of *ppoA* leads to increased resistance to ROS and higher virulence by *Aspergillus* in animal models. Similarly, this model may be applied to barraging cells in *C. parasitica*, as both ROS and secondary metabolite production evidently occur.

### Transcriptome analysis—secondary metabolite biosynthesis

GO terms associated with “Mycotoxin (secondary metabolite) biosynthesis” were also found to be highly enriched in DE genes (see Figure 4). These DE genes included various “tailoring” enzymes involved in adding functional groups and post-transcriptional structural modification during secondary metabolite biosynthesis (P450 monooxygenases, O-methyltransferases, and a peroxidase) (Usui *et al.* 1998; Fujii *et al.* 2001; Qiao *et al.* 2011; Kato *et al.* 2013; Niehaus *et al.* 2013).

**Figure 5:**
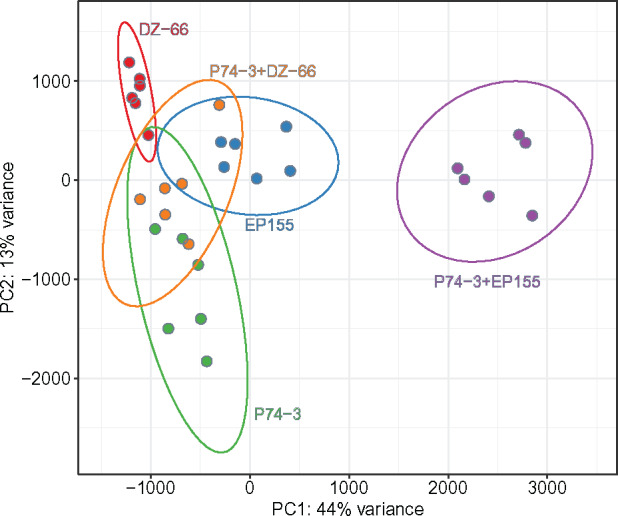
PCA score plot differentiates metabolomes of incompatible co-inoculations (P74-3 + EP155, purple) compared to control samples P74-3 (green), EP155 (blue), DZ-66 (red), and DZ-66 + P74-3 (yellow). Circles represent 95% confidence intervals. Total variance explained in first two PCs is 56.9%.

Mass spectrometry-based metabolomics was used to further explore the involvement of secondary metabolite biosynthesis in barraging cultures during allorecognition. PCA analysis of mass features shows a clear separation of barraging P74-3 + EP155 cultures from control treatments along the *x*-axis or PC1, explaining 43.9% of the variation in the data set (Figure 5). Univariate analysis comparing data from incompatible co-inoculation to monoculture controls and the P74-3 + DZ66 co-inoculation was performed using the R package “muma” (Edoardo *et al.* 2013), which led to the creation of a list of 208 mass features significantly (*P*-value < 0.05) associated with *vic3* allorecognition. Each mass feature represents a discrete *m/z* detected in the HRMS associated with a retention time derived from the chromatography. Many of these mass features represent pseudomolecular ions (protonated, salt adducts, neutral losses, and fragments) associated with a single metabolite and further analysis is required to distill this list into representative or “parent ions.”

It should also be noted that GO term groups associated with secondary metabolite production notably overlap in their gene content with other categories such as “apoptosis,” “mycotoxin biosynthesis,” and “oxidative stress.” Considering the above, activation of genes that have general involvement in secondary metabolite production presents a challenge for further functional analysis. Given a paucity of information in the literature surrounding known secondary metabolites produced by *C. parasitica*, an in-depth metabolomic analysis of the data generated here including annotation of mass feature groups and biosynthetic gene clusters relating to *vic3* incompatibility is beyond the scope of this study.

### Comparison of allorecognition across *vic* loci

**Figure 6:**
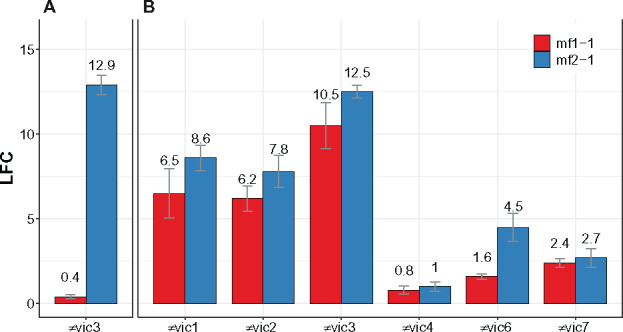
Comparison of differential expression (DE) data acquired by RNA-seq (A, *P*-value < 0.001) and RT-qPCR (panel B, *P*-value < 0.01) of pheromone precursor genes in strains undergoing barrage formation compared to control strains in monoculture. (A) RNA-seq DE data of *mf1-1* (ID: 333020) *mf2-1* (ID: 85578) pheromone gene from *vic3*-incompatible pairing of P74-3 and EP155 (both MAT-2). (B) DE based on RT-qPCR data for *mf1-1* and *mf2-1* genes when EP155 (MAT-2) interacts with strains that are incompatible due to a difference at one of five *vic* loci (*x*-axis). MAT-1 strains with *vic* locus that differs from EP155 in parentheses are P1-5 (≠*vic1*), P1-6 (≠*vic2*), P78-8 (≠*vic3*), P4-4 (≠*vic4*), MJ1-3-20 (≠*vic6*), and P24-33 (≠*vic7*). Whiskers indicate standard error for three replicas (RNA-seq) and at least four biological replicas (RT-qPCR) for each pairing. LFC, log2|fold change|.

Expression of the mating-type pheromone gene, *mf2-1*, drastically increases as a result of *vic3* incompatibility. This is surprising since P74-3 and EP155-derived strains have the same mating type, MAT-2, so a mating interaction is not expected, and yet *mf2-1* demonstrated the highest rate of differential expression in the entire sample set (Figure 3, cluster 1). Upregulation of *mf2-1* is characterized by very low transcript abundance in controls in comparison to very high abundance in barraging cultures. High differential expression was confirmed by RT-qPCR tests with *mf2-1* and this can apparently be used as a marker of *vic3*-associated barrage development (Figure 6B). We used RT-qPCR analysis to further examine *mf2-1* and *mf1-1* mating pheromone expression with each of *vic1*-, *vic2*-, *vic3*-, *vic4*-, *vic6*-, and *vic7*-incompatible pairings using strain pairs that carry MAT-1 and MAT-2 (Supplementary Table S1). Here, *vic1*-, *vic2*-, and *vic3*-associated barrages yielded dramatic increases in expression of *mf2-1* and *mf1-1* (Figure 6B) whereas *vic6*- and *vic7*-associated barrages showed only moderate increases in *mf2-1* and *mf1-1* gene expression. *vic4*-incompatible interaction appears to be uncoupled from mating pheromone gene expression since, similar to monocultures, *mf2-1* and *mf1-1* show almost no change in expression on the 3rd day after co-inoculation of *vic4*-incompatible strains. This may indicate that each type of *vic* incompatibility involves different regulatory networks in *C. parasitica*.

**Figure 7:**
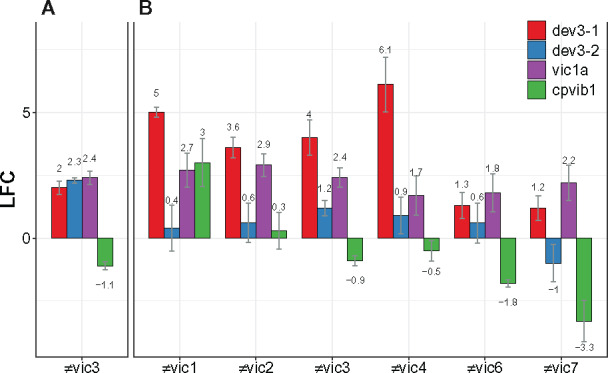
Differential expression of selected HET-domain genes and *cpvib1* gene during different *vic* incompatibilities in relation to monocultures. (A) RNA-seq analysis identified three HET-domain genes as significantly (*P*-value < 0.001) DE during *vic3* incompatibility. (B) RT-qPCR differential expression data (*P*-value < 0.01) of HET-domain genes and *cpvib1* during barrage formation in strain pairs incompatible by single *vic* locus. Gene IDs in *C. parasitica* genome: *dev3-1 *=* *261856, *dev3-2 *=* *262887, *vic1a* = 258862, and *cpvib1 *=* *67224. Strains incompatible with EP155 are P1-5 (≠*vic1*), P1-6 (≠*vic2*), P4-4 (≠*vic4*), P10-18 (≠*vic6*), P24-33 (≠*vic7*), P78-8 (MAT-1), and P74-3 (MAT-2) (≠*vic3*). Whiskers indicate standard error for at least four biological replicates for each pairing (RT-qPCR) or three biological replicates (RNA-seq). LFC, log2|fold change|.

There are six *vic* loci known to trigger incompatibility in *C. parasitica* and only three of these loci, *vic1*, *vic6*, and *vic7*, include genes that encode an HET domain (Cortesi and Milgroom 1998; Zhang *et al.* 2014). Other loci, including *vic3*, do not contain genes encoding an HET domain, suggesting that not all VI responses require HET domain involvement. However, our transcriptome analysis demonstrates that unlinked, nonpolymorphic HET genes can be activated during *vic3*-incompatible reactions and this may be true for other *vic* loci as well. In particular, one HET-domain gene identified as DE during *vic3* allorecognition was *vic1a*, which is part of the *vic1* incompatibility locus (Zhang *et al.* 2014). *vic3*-incompatible strains paired in this experiment, P74-3 and EP155, share the same *vic1a* allele. However, gene *vic1a* was overexpressed in *vic3*-associated barraging samples. In contrast, no genes identified from the *vic2*, *vic4*, *vic6*, *vic7* loci were DE during *vic3*-associated incompatibility. The observed activation of the *vic1a* gene and a subset of other HET domain-containing ORFs present a possible explanation for the absence of a HET domain ORF within the *vic3* locus: it is possible that *vic3* gene(s) function as an upstream activator of *vic1a* and other genetically unlinked HET-domain genes to elicit PCD. To examine the role of a subset of HET-domain genes in other incompatibility settings, we performed a series of RT-qPCR tests (Figure 7). In this experiment, we used different strain pairs, each having different mating types and a difference at one of the *vic* loci (Supplementary Table S1). To our surprise, the *vic1a* gene appeared to be DE under all *vic* incompatibilities. In addition, *dev3-1* showed significant overexpression in strain pairings that were incompatible by *vic1*, *vic2*, *vic3*, or *vic4*, but not for *vic6*- or *vic7*-incompatible pairings. The near-universal pattern of overexpression of *vic1a* and *dev3-1* indicates that there may be a redundancy among HET genes. Furthermore, it is plausible that some HET-domain genes are components that function in several incompatible reactions despite not being genetically linked to the incompatibility locus, and are also not required to be genetically polymorphic. This situation has precedent in the *tol–mat* incompatibility system in *N. crassa* (Shiu and Glass 1999) as discussed above.

How HET-domain genes are controlled at the molecular level is largely unknown aside from examples in *N. crassa* indicating that at least some *het* incompatibility gene expression is modulated by the Ndt80p-like transcription factor VIB-1 (Xiang and Glass 2002). In yeast, the *p53*-like protein Ndt80p is a regulator of meiosis and in *N. crassa* this transcription factor controls multiple functions including female sexual development and formation of ascospores (Hutchison and Glass 2010). Studies with *N. crassa* strains in which *vib-1* is deleted indicate that strains differing at *het-c* are able to form viable heterokaryons (Dementhon *et al.* 2006). There are three paralogs of *p53*-like proteins in the *C. parasitica* genome and none have been previously studied in connection to incompatibility function. The ortholog with the highest similarity to *vib-1*, identified as *cpvib-1* (Cp ID: 67224), showed no significant differential expression during *vic3*-associated barrage (Figure 7). We used RT-qPCR to examine expression levels of *cpvib-1* during barrage in the previously mentioned pairings that are incompatible by single *vic* loci. According to our data, *cpvib-1* showed notable change in expression only in ≠*vic1*, ≠*vic6*, and ≠*vic7* pairings. There seems to be no correlation of *cpvib-1* and the expression of tested HET genes.

### Comparison of VI in *C. parasitica* to HI transcription profiles of *N. crassa* and *P. anserina*

Transcriptional response to HI was previously investigated by microarray analyses in *N. crassa* and *P. anserina* (Hutchison *et al.* 2009; Bidard *et al.* 2013). Both of these previous studies differed from the present study by employing “induced incompatibility techniques,” methods which activate HI factors at permissive temperatures. In the case of *N. crassa*, strains incompatible at the *het-c* locus can form heterokaryons in a temperature-dependent manner if one of the strains carries the mutant allele *pin-c2m* (Kaneko *et al.* 2006). This type of HI in *N. crassa* is referred as *TSinc* (Temperature-Sensitive incompatibility). In this example, strains with *het-c1/pin-c1* can form a heterokaryon with *het-c2/pin-c2m* strains at 34°, showing a low death rate. However, when transferred to a lower temperature (20°) HI is rapidly induced in these heterokaryons. Similarly, in the *P. anserina* model, strains that are incompatible due to nonallelic interactions involving *het-R* and *het-V* present normal culture development at 32° whereas incompatibility reactions occur when these strains are transferred to 26°. One advantage of these systems is that they allow for synchronous induction of alloreconition across the entire mycelium, providing a means to study the precise timing of incompatibility events. In the *C. parasitica* barraging experiment fusion time is not synchronized and cells undergoing nonself fusion likely comprise a small fraction of total mass of mycelium at any given time point. This makes it challenging to separate signals from barraging and non-barraging cells. However, the induced incompatibility technique is restricted to temperature inducible HI systems and may not integrate processes such as cell contact and fusion that occur during natural barrage formation. Temperature-sensitive incompatibility systems are not available for *C. parasitica*. Thus, we used a different technique to analyze incompatibility processes in barraging cells and this should be kept in mind when comparing our results to those from *N. crassa* and *P. anserina*. We are also comparing incompatibility reactions triggered by nonorthologous incompatibility genes in these three species and processes may vary accordingly between these systems.

**Figure 8:**
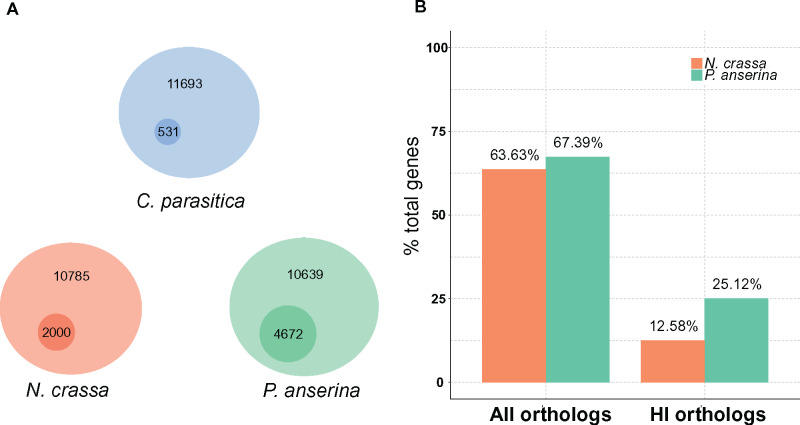
Comparison of incompatibility types in *N. crassa*, *P. anserina*, and *C. parasitica*. (A) Diagram of relative proportion of genes that are DE (log2fold > 2) during VI (*C. parasitica*) and HI (*N. crassa* and *P. anserina*). Large circles represent total number of genes in genomes of each species. Size of the small circles represents proportion of DE genes VI or HI. (B) “All orthologs” represents the proportion of all *C. parasitica* genes (100% on *y*-axis) that have identified orthologs in *N. crassa* or *P. anserina* (*e*-value < 10–10). “HI orthologs” represents the proportion of orthologs that are differentially regulated during HI (*N. crassa* or *P. anserina*), over all genes in *C. parasitica*.

With the above differences in mind, we compared close gene orthologs (BLAST+ $e-\text{value}<10^{-10}$) of *N. crassa* and *P. anserina* from the *C. parasitica* genome (Figure 8). We used the reference genome from *N. crassa* OR74a (Galagan *et al.* 2003) that includes 10,785 protein-coding genes, from which 3447 (32% of total genes in genome) were significantly DE during *het-c*-associated HI (Figure 8A) (Hutchison *et al.* 2009). However, in the *C. parasitica* dataset, we defined DE genes as significant when the DE value was above two (LFC > 2) and, in contrast, some DE values from the *N. crassa* study were below this value. By applying our threshold, we came to use only 2000 (18% of total genes in genome) out of 3447 HI DE genes reported for *N. crassa* (Hutchison *et al.* 2009). Finally, when we select *N. crassa* DE genes, we need to identify how many of them show homology to *C. parasitica* genes overall, and DE genes during *vic3*-incompatibility. As shown in Figure 8B, we identified 6863 or 63% of *C. parasitica* genes that show significant (BLAST threshold $e-\text{value}<10^{-10}$) homology to *N. crassa* genes. Out of the 2000 *N. crassa* HI DE genes, only 1357 (68% of DE HI genes) show homology to *C. parasitica* genes. Similarly, for the *P. anserina* comparison, we used the genome from the S mat+ strain (Espagne *et al.* 2008) that contains 10,588 protein-coding genes. Out of these genes, 4672 (44% of total) were identified as significantly (LFC > 2, *P*-value < 0.001) DE as a result of HI (Bidard *et al.* 2013). Among total *P. anserina* genes, 7170 (67% of total) showed significant homology to *C. parasitica*. Among DE genes during HI in *P. anserina*, 2672 (57% of DE HI genes) showed significant homology to *C. parasitica* (Figure 8B). In comparison to the other two species, *C. parasitica vic3* incompatibility causes differential expression of 531 (4.5% of total) genes. As we can see, induced HI in *P. anserina* and *N. crassa* causes a larger change in gene expression profiles than we observe in barraging *C. parasitica*. This may indicate that induced HI in *N. crassa* and *P. anserina* result in a larger impact on fungal cells compared to strains undergoing barrage or that differences in methodologies (discussed above) obscure some DE genes in *C. parasitica*. Furthermore, pairwise correlation analyses among DE HI genes between *C. parasitica* and *N. crassa* (*P*-value = 0.1) or *C. parasitica* and *P. anserina* (*P*-value = 0.3) was not statistically significant. However, there is a strong correlation between *N. crassa* and *P. anserina* HI genes DE during HI [calculated *P*-value < 10^−6^ in this study and *P*-value < 10^−4^ by Bidard *et al.* (2013)].

Even considering these differences, we can point out a few similarities between *C. parasitica* and the other two HI responses at the transcript level. There are five orthologs that are DE in all three species during incompatibility reactions (Table 2). These five genes represent some of the processes we already described: secondary metabolism, cell death, and growth. One gene universally downregulated in all three species is an ortholog of the Arabidopsis neutral ceramidase (see Table 2; IDs: 100328, NCU04721, Pa_4_6950). This protein catalyzes degradation of ceramide to sphingosine. Various studies on tumor cells in animals showed that ceramide promotes apoptotic signals coming from tumor necrosis factor (TNF) receptors (Obeid *et al.* 1993). Sphingosine, on the other hand, was shown to inhibit apoptosis and promotes cell growth in animals (Ohta *et al.* 1994). Also, a previous study indicated that activity of ceramidase in combination with growth factors promotes growth of human fibroblast cell culture (Coroneos *et al.* 1995). Additionally, it was shown that induction of ceramide can downregulate cytochrome p450 2C11 in mice, a process that mimics Interleukin-1 signaling (Nikolova-Karakashian *et al.* 1997). Downregulation of cytochrome p450s by Interleukin-1 activation is proposed to be part of the immune response making rat cells more prone to death by decreasing defence mechanisms (Chen *et al.* 1995). As we showed previously, p450 proteins are enriched among DE genes and are mostly upregulated during barrage (see Figure 4 and Supplementary Table S1). Thus, we speculate that downregulation of ceramidase during HI may inhibit growth processes and favor apoptosis-like response in barraging cells.

**Table 2:** Common orthologs DE in *C. parasitica* during *vic3* associated barrage, and *N. crassa* and *P. anserina* during HI

Cp ID	Cp LFC	Pa ID	Pa LFC	Nc ID	Nc LFC	UniProt ID	Putative protein Function
254007	5.4	Pa_0_160	3.79	NCU02031	−2.09	Q12587	Cytochrome P450
263261	3.9	Pa_1_6810	2.83	NCU07474	−2.69	Q4WZ69	Festuclavine dehydrogenase
337702	3.1	Pa_1_5200	−3.38	NCU00821	−2.17	P39932	Sugar transporter STL1
358581	1.9	Pa_2_2300	−2.69	NCU01258	−3.10	Q871Z4	Cyanate hydratase (Cyanase)
100328	−2.4	Pa_4_6950	−2.88	NCU04721	−2.28	Q304B9	Neutral ceramidase (N-CDase)

Three out five genes in the list are associated with the modification of secondary metabolite scaffolds (as tailoring enzymes) or detoxification systems. An ortholog of cyanase from *N. crassa* (Table 2) is upregulated during barrage in *C. parasitica*. Cyanase is a detoxification enzyme that converts cyanate to NH_3_ and CO_2_ (Elleuche and Pöggeler 2008; Elmore *et al.* 2015). The other two genes show homology to fungal festuclavine dehydrogenase (an oxidoreductase) (Coyle and Panaccione 2005) and cytochrome p450, both possibly involved in secondary metabolite modification. All three genes are upregulated in *C. parasitica* and DE (either up or down) in *P. anserina* and *N. crassa*, which may indicate strong activation of secondary metabolism during normal allorecognition reaction compared to temperature-induced HI.

Among upregulated genes, one functionally similar group worth noting are the genes involved in detoxification. As a particular example, the ortholog of GST gene (Cp ID: 58765) is one of the most highly upregulated genes during incompatibility in *P. anserina* and *C. parasitica*. This detoxification protein plays an anti-apoptotic function in neutralizing toxic compounds (Circu and Aw 2012). Considering this, it is no surprise to see cyanase and GST overexpressed during barrage, which may represent an increased detoxification signal in the *C. parasitica* system that originates in “non-barraging” cells exposed to the toxic environment produced by barraging hyphae. Such nonbarraging cells are presumably not present in the *N. crassa* and *P. anserina* temperature-induced systems.

Cell death mechanisms associated with HI seem to be distinct from known yeast PCD processes (Madeo *et al.* 2002; Severin and Hyman 2002; Wissing *et al.* 2004; Pozniakovsky *et al.* 2005; Zhang et al. 2006; Carmona-Gutierrez *et al.* 2010). Previous analysis showed that orthologs of 11 yeast apoptosis genes are not activated during HI-associated PCD in *N. crassa* (Hutchison *et al.* 2009). Disruption of similar genes in yeast leads to resistance to apoptotic death (Khan *et al.* 2005; Guaragnella *et al.* 2006), but in the case of *N. crassa*, disruption of two metacaspases (NCU09882, NCU02400) and AIF (apoptosis-inducing factor, NCU05850) does not impede HI-associated PCD (Hutchison *et al.* 2009). Eight of these 11 *N. crassa* genes have identifiable orthologs in the *C. parasitica* genome, but none are differentially regulated during the barrage process (data not shown). These genes include orthologs of two metacaspases (NCU09882, NCU02400), Cytochrome *c* (NCU01808), Ste4p (Cp ID: 105373, Nc ID: NCU00440), G-protein beta-subunit (NCU00440), Lag1 (NCU00008), Ppa1p (NCU09747), and HSP70 (NCU09602) (Hutchison *et al.* 2009). Similarly, other apoptotic genes identified in yeast do not show differential expression during barrage in *C. parasitica*.

A common feature in *C. parasitica* barrage and *N. crassa* induced HI is the production of ROS. In *N. crassa*, genes involved in ROS response, such as Cytochrome *c*, NADPH oxidase, and glutaredoxin are overexpressed during HI (Hutchison *et al.* 2009). In *C. parasitica*, none of these genes are DE in barraging strains. However, this does not indicate a lack of ROS accumulation in *C. parasitica* during barrage formation. On the contrary, stress response proteins like GST and QO (PIG3, see Table 1, ID: 333952) show increased expression during barrage and staining with DCFDA, an ROS indicator, confirmed ROS production in barraging cells, similar to observations from *N. crassa* (Hutchison *et al.* 2009) (see Figure 1C). As far as we know, no comparable experiments were performed for *P. anserina*. However, protein domains related to GST, QO, Cytochrome p450 proteins were found to be abundantly upregulated in *P. anserina* HI strains (Bidard *et al.* 2013). These data suggest that production of ROS and associated PCD mechanisms present a common feature of nonself recognition among filamentous fungi.

Lastly, the upregulation of HET-domain genes was identified in the transcriptome data sets for all three species. *Cryphonectria parasitica* and *N. crassa* showed very similar proportions of activated HET-domain genes (Hutchison *et al.* 2009). There were only five HET-domain genes DE in the *N. crassa* dataset, but surprisingly, four of them were downregulated. The one upregulated HET gene is a hypothetical protein (NCU03507) showing similarity to *het-6* (NCU03533) and appears as upregulated 1 h after HI induction (temperature decrease). The remaining four downregulated genes were *het-6OR* (NCU03533), *het-c1* (NCU03125), *het-c2* (NCU07947), and another uncharacterized HET-domain gene (NCU09045). Both *het-c1* and *het-c2* are alternate alleles from the *het-c* locus that are known triggers for HI (Kaneko *et al.* 2006; Hutchison *et al.* 2009). In contrast, out of 130 predicted HET-domain genes in the *P. anserina* genome, more than 50% are activated during induced HI. Even though *C. parasitica* and *N. crassa* have a smaller number of DE HET-domain genes compared to *P. anserina*, all three species demonstrate similarities in their incompatibility reactions. As described previously, HET-domain genes are only known as activators of incompatibility reactions, and activation of a single HET gene may be enough to produce the entire spectrum of incompatibility symptoms (Paoletti and Clavé 2007). Thus, in our analysis, we can additionally speculate that the number of HET-domain genes activated during barrage or HI may not influence the severity of an incompatibility reaction.

## Discussion

In this study, we performed a transcriptome analysis to identify underlying molecular processes of *vic3*-associated incompatibility in *C. parasitica*. From the analysis of DE genes, we infer that at least five major processes are activated during barrage formation: apoptosis, detoxification (against ROS and secondary metabolites), pheromones synthesis, RNA interference, and allorecognition (HET-domain genes).

Of these general processes, overexpression of pheromone genes during barrage was surprising for at least two reasons. First, previous studies with *C. parasitica* indicate that pheromone genes are constitutively expressed (Kazmierczak *et al.* 1996; Turina *et al.* 2003), whereas we provide evidence that pheromone genes are upregulated during *vic3*-associated incompatibility. Second, the drastic increase in expression of genes involved in sexual reproduction during VI is unexpected given that VI is not considered part of the sexual cycle. In addition, we would not expect sexual reproduction signaling during interaction between EP155 (nor DZ-66) and P74-3 given that these strains are of the same mating type (MAT-2). A connection between asexual sporulation and activation of genes involved in sexual process has not been observed in filamentous fungi and it may be that any such connection is genus or species specific. A closer look at the *C. parasitica* life cycle shows that it does not involve development of specialized male sexual sporangia. Instead, *C. parasitica* uses asexual conidia as male gametes (Marra and Milgroom 2001). Thus, we can suggest inhibition of the sexual cycle happens downstream of mating genes and pheromones. Interplay between sexual and asexual sporulation has been demonstrated to involve *ppoA* from *Aspergillus nidulans* (Tsitsigiannis *et al.* 2004). PpoA is a fatty acid dioxygenase that is involved in regulation of balance between anamorph and teleomorph stages of development. Deletion of *ppoA* causes a shift toward increased asexual spore production. Downregulation of *ppoA* (ID: 332509) provides a plausible mechanism by which the fungus is able to inhibit the sexual cycle during barrage formation even while some mating genes are activated.

Additional evidence of association of pheromone genes with asexual sporulation comes from RT-qPCR data for other incompatibility types. In Figure 6, we can see that *vic6*-, *vic7*-, and especially *vic4*-associated barrages show moderate or complete lack of pheromone gene differential expression. In the case of *vic4*, we can suggest that barrage here is uncoupled from mating pheromones. Thus, considering our previous interpretation of the role of pheromones in barrage, we suggest that a lack of pheromone expression indicates low levels of conidiation and weak barrage reaction. This is consistent with previous observations that, out of the six characterized *vic* loci in *C. parasitica*, *vic4* stands out as having a “weak” barrage phenotype that allows 100% hypovirus transmission (Cortesi *et al.* 2001) and does not prevent heterokaryon formation (Smith *et al.* 2006).

There is no known direct connection on a molecular level between HET-domain genes and PCD. However, a plausible link of activation of HET-domain genes and PCD could occur concomitantly with the activation of genes involved with secondary metabolism as previous observations suggested that HI-associated PCD in fungi resembles basic defence reactions in animals and plants (Lam *et al.* 2001). Our transcriptome and metabolomics data indicate that rapid activation of detoxification mechanisms coincide with overexpression of genes involved in secondary metabolism. Our analyses indicated that secondary metabolism is triggered by incompatibility and we posit that this is a probable cause of PCD in *C. parasitica*. A majority of stress genes identified in this study are known to be activated in response to high levels of ROS as a result of apoptosis (Ray *et al.* 2012). This leads us to suggest that barrage formation may involve several PCD pathways. Activation of secondary metabolite production, detoxification pathways, and ROS levels point to heterogenic structure of barrage. Hyphae that undergo incompatible fusion represent a small minority and may in fact not undergo intrinsic PCD but produce signal molecules or toxins that trigger extrinsic PCD in neighboring cells.
